# Comprehensive Large-Scale Integrative Analysis of Omics Data To Accelerate Specialized Metabolite Discovery

**DOI:** 10.1128/mSystems.00726-21

**Published:** 2021-08-24

**Authors:** Joris J. R. Louwen, Justin J. J. van der Hooft

**Affiliations:** a Bioinformatics Group, Wageningen University, Wageningen, the Netherlands

**Keywords:** computational biology, computational metabolomics, data mining, genomics, integrative omics, mass spectrometry, microbiome, natural products, specialized metabolites

## Abstract

Microbial specialized metabolites are key mediators in host-microbiome interactions. Most of the chemical space produced by the microbiome currently remains unexplored and uncharacterized. This situation calls for new and improved methods to exploit the growing publicly available genomic and metabolomic data sets and connect the outcomes to structural and functional knowledge inferred from transcriptomics and proteomics experiments. Here, we first describe currently available approaches that support the comprehensive mining of metabolomics and genomics data. Next, we provide our vision on how to move forward toward the automated linking of omics data of specialized metabolites to their structures, biosynthesis pathways, producers, and functions.

## COMMENTARY

Microbially produced and metabolized small molecules are everywhere: in the soil, plants, microbes, and our body. They constitute many functions ranging from simply providing nutrition to more specialistic tasks such as conveying messages or selectively killing organisms. These microbial specialized metabolites have been instrumental for humankind in medical applications such as antibiotics. The emerging threat of antimicrobial resistance is challenging our current medical advances. This has sparked a renewed interest in mining and elucidating the microbiome chemical diversity to find bioactive molecules.

The four main omics technologies are increasingly used to study microbial chemistry present in natural extracts. Advanced genome mining provides us with an organism’s biosynthetic potential, while transcriptomics and proteomics allow insight into pathway activity through the regulation of transcript and protein levels. Finally, untargeted tandem mass spectrometric (MS/MS) metabolomics records mass spectral data for many microbial natural products. Today, the comprehensive study of the microbial specialized metabolome is mainly hampered by our ability to structurally and functionally annotate omics features.

Technical, analytical, and software advances in the four omics technologies have been impressive over the last 2 decades, yet their integrated analysis remains very challenging. Thus, it is still difficult to rapidly assess the novelty of a metabolite, find the organism that produces it, and learn its function within an ecosystem (1). The Integrated Omics for Metabolomics and Genomics Annotation (iOMEGA) project (see https://github.com/iomega and https://www.esciencecenter.nl/projects/integrated-omics-analysis-for-small-molecule-mediated-host-microbiome-interactions/) led by our group enabled us to explore the current obstacles and opportunities to first improve these omics pillars separately and then build connections to link producers to molecular products (1).

In this perspective, we highlight our contributions to the emerging field of computational metabolomics, how these developments are foundational to performing integrated omics analyses, and how they will accelerate natural product discovery through improved structural and functional annotation of omics profiles.

Metabolome mining tools have been developed that mostly use the collection of MS/MS spectra (or election impact spectra for volatiles or derivatized metabolites [2]) as a representative of natural extracts. Alongside, repositories have emerged to archive the annotated spectra or spectral patterns that these mining tools recognize (3–5). In addition, multiple tools have appeared that mine genomes for biosynthetic gene clusters (BGCs) (6, 7), and precomputed mining results for all publicly available genomes are now also available for large-scale analyses. Experimentally characterized BGCs linked to structural information can be stored in a dedicated repository (8).

**FIG 1:**
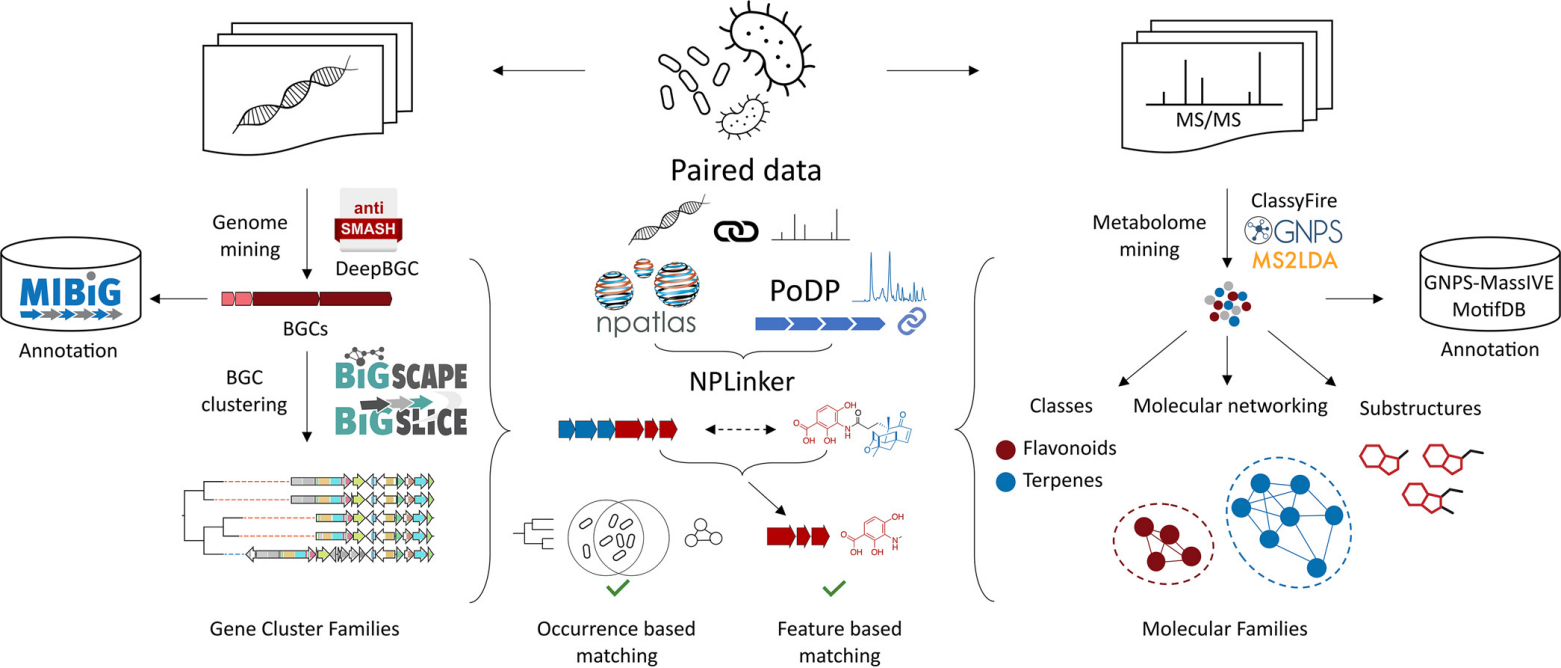
Current state-of-the-art ecosystem of genomics (left) and metabolomics (right) natural product research, brought together by paired omics approaches (middle). Genomes are mined for biosynthetic gene clusters (BGCs) through tools such as antiSMASH and DeepBGC, and BGCs with structurally characterized products are stored in databases like MIBiG. BGCs are clustered into families with BiG-SCAPE and BiG-SLiCE. To infer compound classes, molecular families, and substructures, metabolomes (represented by collections of MS/MS spectra) are mined with tools such as ClassyFire, GNPS, MS2LDA, and MolNetEnhancer. Structural annotations relevant for microbiome research are stored in databases such as NP Atlas and MotifDB, and reference spectra are available in repositories such as GNPS-MassIVE. Paired data stored in platforms such as the Paired Omics Data Platform (PoDP) combine the two sides, which facilitates multi-omics approaches such as NPLinker that links gene cluster families (GCFs) to molecular families (MFs) through sample occurrence (also known as strain correlation) and feature-based matching.

In currently existing omics annotation workflows (Fig. 1), matching to repositories is the most reliable step to add structural information to metabolomics profiles enabling biochemical interpretation. Moreover, structure databases with well-curated (meta)data (i.e., first isolation paper, validated biosynthetic gene cluster, and complete and computer-readable structural information, etc.) are also key to enable the accurate annotation of omics profiles with microbial metabolites (3, 8, 9). While increasing numbers of reference spectra and validated BGCs are deposited in public repositories, the resulting rates of matching to omics profiles remain low, and the elucidation of full structures thus remains very challenging. This has sparked the recent development of other approaches based on substructure-based, chemical compound class-based, and network-based techniques, which are all highlighted below.

Substructure-based metabolomics workflows use the idea that the basic building blocks that are shared by different naturally occurring structures will yield similar spectral signals. It is now possible to mine for substructure patterns in metabolomics profiles and store annotated patterns in a repository for reuse in future experiments (4, 5). For example, annotated substructures of *Salinispora* and *Streptomyces* bacteria are now available to accelerate substructure analysis of bacterial extracts from related strains.

Chemical compound class annotations can also provide useful information about metabolites that can be used to obtain a high-level overview of the type of chemistry present in natural extracts. For example, specific compound classes such as macrolides or lanthipeptides are likely to be microbially derived. In both genomics and metabolomics workflows, tools have emerged to assign chemical compound class information to BGCs or mass spectra (6, 10).

Network-based analysis is beneficial as it facilitates the large-scale analysis of BGC and MS/MS spectrum ensembles by grouping them into families (3, 11, 12) and allows the propagation of spectral annotations within molecular families. Various approaches to capture structural information at the structural, chemical class, and substructure levels have emerged, and for metabolomics data, MolNetEnhancer (10) was the first tool to integrate and visualize all that information in one place.

Multi-omics approaches facilitate structural and functional annotations by combining complementary information about microbial chemistry. Paired data sets are needed to perform integrative omics mining analysis (1). Recently, the Paired Omics Data Platform (PoDP) was developed, which already holds >4,800 links between (meta)genomes and metabolomics data sets (13). This will allow the detection of new links between BGCs, MS/MS spectra, and compounds, for example, through platforms such as NPLinker that facilitate the computation of various strain correlation-based and feature-based linking scores (1, 14) (Fig. 1).

Looking into the future, based on early successes in omics analysis (4, 7, 15), we envision that machine learning (ML) algorithms will become increasingly important. For example, in metabolomics analysis, mass spectral similarity metrics play a pivotal role across many tasks, including library matching and analogue searching. Our group applied ML to this task for the first time, resulting in the unsupervised Spec2Vec algorithm (16), which showed increased performance in library matching and analogue searching through the learning of relationships between mass features in many MS/MS spectra. Furthermore, we recently proposed the supervised MS2DeepScore algorithm (17), which was trained to learn molecular structural similarities based on MS/MS spectral pairs, resulting in an even better overall performance.

**FIG 2:**
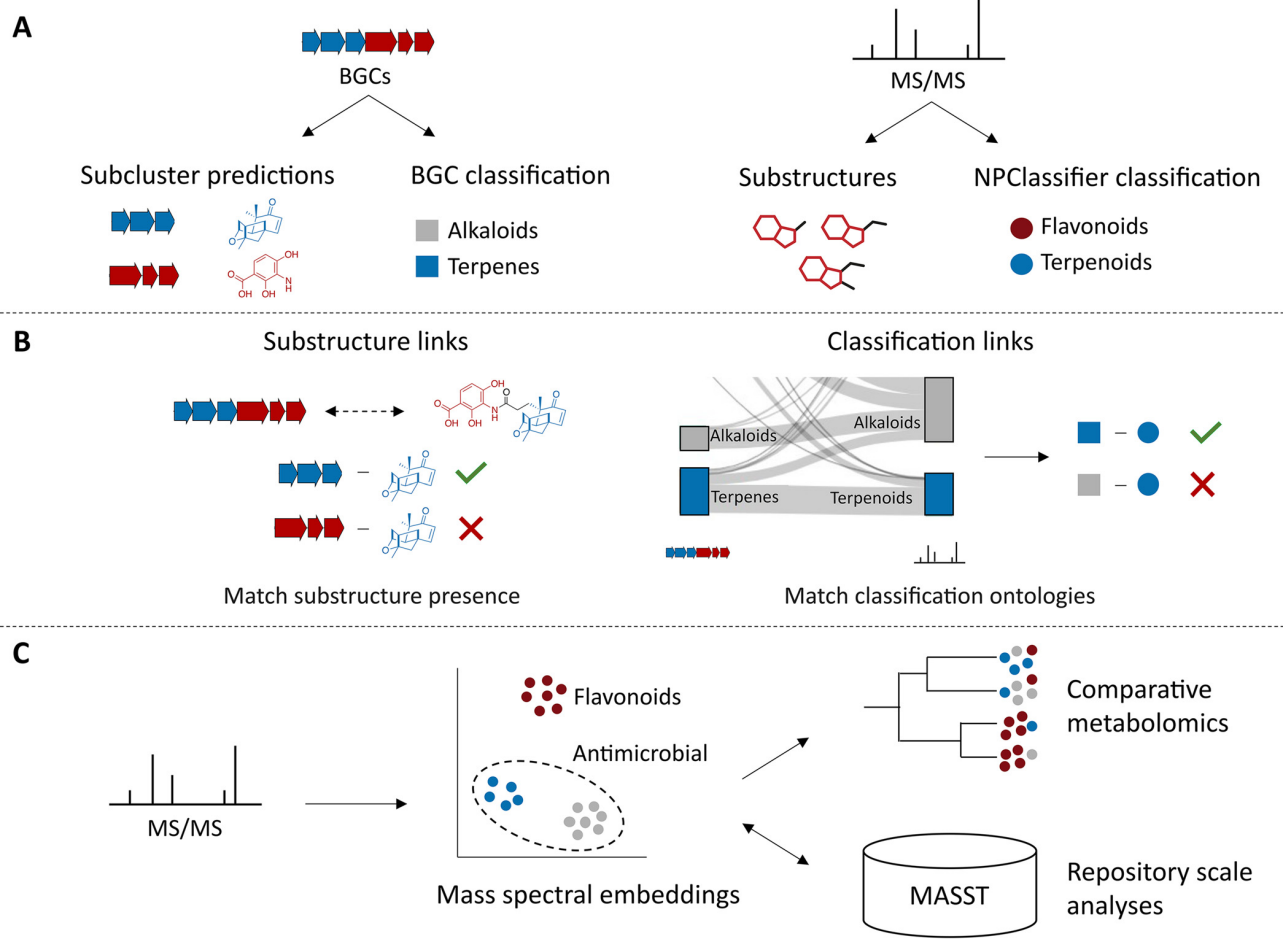
Current and envisioned advances in multi-omics natural product discovery research. (A) Improved detection of subclusters and relevant natural product-related chemical compound classes in BGCs and MS/MS spectra will become possible based on machine learning-based computational tools. (B) We envision combining the existing BGC-metabolite matching approaches with substructure and chemical class predictions in platforms such as NPLinker. NPClassifier is a novel ML-based class predictor that considers both structural features and historical relationships between metabolites as defined by natural product researchers. (C) Mass spectral embeddings learned by Spec2Vec and trained with MS2DeepScore will enable fast and improved spectral similarity scoring. The bases for these mass spectral embeddings are the relationships between mass fragments and neutral losses based on their presence/absence in a large set of mass spectra. We expect that these embeddings will allow the rapid annotation of classes, substructures, or other labels such as pathways or functions based on clustering techniques. Finally, the developed workflows can also form the basis for improved comparative and repository-wide metabolomics approaches that highlight shared and novel chemistry produced by microbiomes.

We expect that the learned unsupervised and trained supervised mass spectral embeddings to compute these novel similarity metrics will serve as the input for novel scores to facilitate integrated omics analysis in the recently established NPLinker platform (14). Furthermore, where existing annotation pipelines often struggle for sizable specialized metabolites, analyses based on these mass spectral embeddings are fast, scalable, and thus compatible with an integrated analysis framework for natural products (Fig. 2). Here, it is noteworthy that ML also allowed the development of the natural product-compatible structural classification scheme NPClassifier, which considers structural, functional, and biosynthetic relationships as historically defined by natural product researchers (18).

In integrative omics for natural product discovery, one of the central aims is the linking of BGCs with the MS/MS spectra of the products that they encode, to facilitate the structural elucidation of the metabolite product(s), establish the producer(s), and infer the function of the specialized metabolites through annotated genes neighboring the BGC. We hypothesize that metabolite annotations can be used to improve the linking of BGC and metabolome information (Fig. 2). By comparing chemical compound classes with BGC classes, it would be possible to rerank BGC-MS/MS links based on the likelihood of occurrence, thereby removing implausible links such as a peptidic compound being produced by a terpene BGC. Similarly, we think that links could be reranked based on shared substructure content inferred from metabolomics and genomics data. Substructures can be annotated by metabolome mining tools from MS/MS spectra and predicted from BGCs by identifying subclusters, which can currently be done through either a targeted or a statistical approach (19). We anticipate that ML approaches for subcluster detection will further facilitate this.

To understand the function of specialized metabolites, comparative analyses between multiple relevant conditions or phenotypes and the linking of functional information inferred from transcriptomics or proteomics experiments will be key. To support such analyses, metabolome mining workflows were linked to statistical approaches through the coupling of metabolite feature recognition tools (20), even in a chemically informed manner (21). When grouped in metabolic pathways or metabolite sets, comparative analyses at the pathway activity level linked to BGC abundance profiles from (meta)transcriptomics can yield further information about which functional pathways or metabolite groups specialized metabolites are part of. To facilitate such analyses in the future, recording expression data through transcriptomics or proteomics in paired data repositories like the PoDP will be essential.

With vastly growing public databases, repository-scale analyses become increasingly relevant to assess the novelty of discovered metabolites by comparing experimental omics profiles not only to validated data (i.e., BGCs and MS/MS spectra assigned to metabolite products) but also to data from all publicly available omics profiles (22, 23). We envision that ML-based (and in particular mass spectral embedding-based) approaches will accelerate current approaches even further (24). It is important to realize that for reliable omics annotations and comparative analyses, consistent and curated metadata are key, for example, in the form of a controlled vocabulary for metabolomics metadata (25) and BGC metadata (8).

We expect that in the near future, the above-described toolset will become more accurate and user-friendly. Microbiome and natural product researchers will then be able to rapidly prioritize novel chemistry in omics profiles. Through accurate genome-metabolome linking, the genetic machinery and mass spectral data will be easily connected. This will boost the structural elucidation of novel metabolite products and enable the recognition of their producers in complex communities such as those originating from soil or our gut. This in turn will allow researchers, i.e., through functional omics profiling and BGC-neighboring gene annotations, to select potential novel antibiotics in their samples, e.g., based on resistance-associated annotations. We anticipate that such applications will help to combat the currently looming antimicrobial resistance pandemic.

To conclude, advances in computational metabolomics and genome mining have enabled natural product-targeted multi-omics analyses, and tools are starting to be in place to exploit recorded paired data sets and annotate omics profiles with structural and functional information to accelerate natural product discovery.
